# Resistance intensity test (RIT): a novel bioassay for quantifying the level of acaricide resistance in *Rhipicephalus microplus* ticks

**DOI:** 10.1186/s13071-024-06561-6

**Published:** 2024-11-20

**Authors:** Frans Jongejan, Laura Berger, Laura Homminga, Iris Hulsebos, Alita Petersen, Priscila Teixeira Ferreira, José Reck, Guilherme Klafke

**Affiliations:** 1 Department of Veterinary Tropical Diseases, Faculty of Veterinary Science, University of Pretoria, Private Bag X04, Onderstepoort, 0110 Republic of South Africa; 2 TBD International BV, BioScience Center, Runderweg, 6,8219 PK Lelystad, The Netherlands; 3 Instituto de Pesquisas Veterinárias Desidério Finamor, Estrada Do Conde, 6000, Eldorado Do Sul, RS 92990-000 Brazil

**Keywords:** Resistance intensity test, Adult immersion test, Larval packet test, Acaricide resistance, Ticks, Cattle, Brazil

## Abstract

**Background:**

One bioassay for detecting acaricide resistance in livestock ticks is the adult immersion test (AIT), wherein engorged ticks are briefly immersed into a solution of a particular acaricidal compound and examined for mortality, their egg-laying capacity and offspring hatchability in vitro. Usually, the recommended label dose or an established discriminating dose of an acaricide is used to determine high mortality (≥ 95%) of susceptible tick strains. Such a test intends to detect the presence of resistance in a tick population. However, the adult immersion test does not directly translate the bioassay results to the predicted efficacy in the field. In this paper, we used the AIT as an initial screening bioassay supplemented with the resistance intensity test (RIT), a novel larval-based bioassay, wherein the resistance level can be determined and translated to the expected field efficacy. This was done by adopting World Health Organisation (WHO) guidelines for resistance detection in mosquitoes, which combines a 1 × recommended dose with 5 × and 10 × concentrated doses to reveal low, moderate and high resistance intensity, respectively.

**Methods:**

Engorged *Rhipicephalus microplus* ticks were collected from cattle at six different ranches across Rio Grande do Sul, Brazil, as part of the state’s acaricide resistance surveillance program. Groups of adult ticks from each field collection were subjected to the AIT from each field sample. Additionally, engorged female ticks from each ranch were allowed to lay eggs, and their larval progeny aged 14 to 28 days were then used in the RIT. Deltamethrin and a combination of cypermethrin, chlorpyrifos and piperonyl butoxide were used in both tests, and the results were statistically analysed.

**Results:**

The in vitro efficacy of deltamethrin against adult ticks in the AIT ranged between 8.74% and 25.38%. The corresponding RIT results on their larval progeny indicated a high resistance level. In the immersion test, the in vitro efficacy of the combination of cypermethrin, chlorpyrifos, and piperonyl butoxide against adult ticks ranged between 49.31% and 100%. The corresponding RIT results on their larval progeny indicated a similar response ranging from fully susceptible to low or moderate resistance. The Pearson correlation coefficient (*r* = 0.883) showed a high correlation between tick mortality at the 1 × recommended concentrations of acaricides in both tests.

**Conclusions:**

The resistance intensity test is a valuable addition to the range of bioassays currently available for detecting acaricide resistance by determining the level of acaricide resistance. This is relevant to whether or not to continue using a particular acaricidal class for controlling cattle ticks.

**Graphical Abstract:**

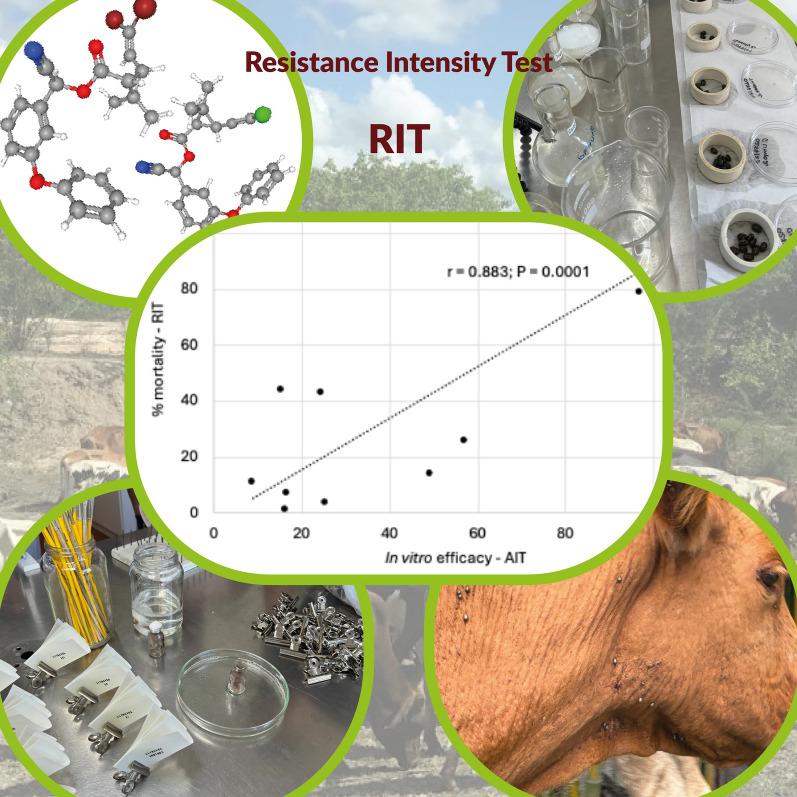

## Background

Ticks infesting cattle constitute a significant constraint to animal production in tropical and subtropical regions through the adverse effects of tick feeding and indirectly through the transmission of tick-borne pathogens [1, 2]. *Rhipicephalus microplus* (Canestrini, 1887), a highly invasive tick, is the most important cattle tick widely distributed in Asia, sub-Saharan Africa and South and Central American cattle production areas. This tick is also Brazil’s most important ectoparasite of livestock, causing losses exceeding three billion US dollars, as estimated in 2014 [3]. Tick control depends almost exclusively on applying acaricidal compounds to cattle, which has resulted in the selection of resistant tick populations [4–6]. As a result, acaricide resistance in ticks is a significant concern for the livestock industry in Brazil and other cattle-producing areas [6]. The situation in Brazil has been described as a worst-case scenario in the world, where resistance has been reported against six of seven classes of acaricides (i.e. organophosphates, formamidines, synthetic pyrethroids, macrocyclic lactones, phenylpyrazoles and benzoylphenyl ureas) [7, 8].

In Rio Grande do Sul, Brazil, cattle carry a significant *Bos taurus* genetic trait, resulting in a higher prevalence of tick infestation and susceptibility to tick-borne diseases. Moreover, their early maturity and enhanced meat quality have led to an upsurge in crossbreeding and the expansion of taurine breeds to other Brazilian regions [9]. This trend contributes to an increased susceptibility of beef cattle to ticks across Brazil [10]. The acaricide resistance status of cattle ticks in Brazil is monitored by conducting in vitro laboratory bioassays. The Food and Agriculture Organization (FAO) of the United Nations recommends using the larval packet test (LPT) for acaricide resistance diagnosis in cattle ticks, as mentioned in their 2004 guidelines [11]. The method was adapted from Stone and Haydock’s technique which exposed tick larvae to filter paper packets impregnated with the technical grade active ingredients dissolved in olive oil [12]. The value of the LPT lies in providing a resistance ratios index on the basis of the ratio between the lethal concentration for 50% of the population (LC50) of the population to be tested and a reference susceptible tick strain. However, LC50 ratios are difficult to translate into practical advice for farmers. The larval immersion test (LIT) can also determine the LC50 of acaricides. It has been shown that the LIT is more sensitive than the LPT for diagnosing resistance to macrocyclic lactones [13]. Therefore, using the LPT and LIT in Brazil for resistance detection is restricted to only a few specialised laboratories that mostly use it in epidemiological surveys [9].

FAO also recommends using the adult immersion test (AIT) for resistance detection and monitoring in livestock ticks [11]. AIT, initially described by Drummond et al. in 1973 [14], is a bioassay wherein engorged female ticks are immersed in acaricide solutions and subsequently monitored under laboratory conditions for their capacity to produce eggs and their hatchability. Currently, AIT is the primary diagnostic method employed in most federal and state research institutions in Brazil to assess acaricide resistance to ticks [9]. The AIT is often used as an assay with a single dose of acaricide rather than a test using various concentrations of drugs. It is widely used as a screening assay to determine the acaricidal in vitro efficacy for commercial acaricides. It also informs farmers and practitioners on the expected efficacy of an acaricide formulation when used in the field. Moreover, AIT can also be used for products wherein different classes of acaricides are combined (e.g. cypermethrin/chlorpyrifos) since there is no standardised LPT for detecting resistance in these mixtures that is commonly used in Brazil. However, although most laboratories in Brazil utilise AIT to assist veterinarians/producers in selecting an effective acaricidal product, it is often difficult to extrapolate bioassay results into concrete advice to mitigate acaricide resistance in the field.

This study was conducted to provide novel options for the decision-making process regarding acaricide selection, giving more information regarding the intensity of resistance and its potential impact on tick control strategies. In this paper, we used the AIT as an initial screening bioassay supplemented with the resistance intensity test (RIT), a novel larval-based bioassay, wherein the resistance level can be easily determined. This was done by adopting World health Organisation (WHO) guidelines for resistance detection in mosquitoes, which combines a 1 × recommended dose with 5 × and 10 × concentrated doses to reveal low, moderate and high resistance intensity, respectively. This reduces the number of laboratory tests and provides relevant information on the resistance of livestock ticks on cattle at the ranch level.

## Methods

### Ticks

Field samples of *R*. *microplus* were collected in November and December 2023. Engorged female ticks from six different ranches across the state of Rio Grande do Sul, Brazil, were submitted to the Laboratory of Parasitology at the Instituto de Pesquisas Veterinárias Desidério Finamor (Eldorado do Sul, Brazil) by farmers or veterinarians. These submissions were part of the state’s acaricide resistance surveillance program, which involves conducting laboratory tests to monitor resistance levels. Cattle farmers and veterinarians were instructed to send the ticks following the recommendations of FAO [11]. Each sample contained approximately 200 ticks collected from between 5 and 10 animals and was received within 48 h of collection from cattle on the respective farms.

### Preparation of ticks

Tick handling procedures followed the guidelines recommended by FAO [11]. Upon arrival, the ticks were washed with water, dried with paper towels and selected on the basis of size (> 8.0 mm), absence of injuries, and vitality. Selected ticks were placed in Petri dishes (15 cm diameter, 2 cm depth) for further processing in the bioassays. A group of 90 ticks from each sample was used directly in the AIT. Additionally, 30 engorged female ticks from the same sample were transferred to a Petri dish (90 mm × 10 mm) and incubated in an environmental chamber set to 27 ± 1 °C with 80–90% relative humidity for 14 days to allow for egg laying. After egg production, the eggs were pooled and incubated in 5 ml serum vials sealed with cotton plugs under the same conditions to permit larval hatching. Larvae aged 14–28 days were subsequently used in the RIT.

### Adult immersion test (AIT)

To evaluate the acaricidal activity against adult ticks, we followed the adult immersion test (AIT) protocol as described by Drummond et al. [14], with slight modifications. Acaricide solutions were prepared by diluting the products according to the label recommendations for cattle treatment via spraying. The preparations were as follows: (i) deltamethrin (Butox^®^ P CE25, MSD Saúde Animal, São Paulo, Brazil) at a 1:1000 dilution (final concentration: 25 mg/ml) and (ii) a combination of cypermethrin, chlorpyrifos, and piperonyl butoxide (Cyperclor Plus^®^, Ceva Saúde Animal Ltda, Paulínia, Brazil) at a 1:800 dilution (final concentrations: 18.75 mg/ml, 31.25 mg/ml and 18.75 mg/ml, respectively). Groups of ten engorged female ticks, sorted by weight to ensure homogeneity, were immersed in 100 ml of the acaricide solution for 5 min. Control groups were treated similarly but immersed in distilled water. After immersion, the ticks were drained using a metal sieve, blotted dry with paper towels, and placed in plastic Petri dishes (90 mm × 10 mm). These dishes were then incubated in an environmental chamber set to 27 ± 1 °C with 80–90% relative humidity for 14 days to allow for egg production. Then, the eggs from each dish were weighed and transferred to 10 mL glass assay tubes sealed with cotton plugs and incubated under the same conditions for 4 weeks to allow larval hatching. After this period, the percentage of larval hatching was visually estimated using a stereomicroscope, comparing the proportion of empty eggs to the total egg mass. The same operator conducted all evaluations to ensure consistency. The experiments were performed in triplicate, with three independent groups of ten female ticks exposed to freshly prepared solutions for each acaricidal treatment and control group.

### Resistance intensity test (RIT)

The resistance intensity test was prepared with commercial deltamethrin (Vectocid^®^) and Vectoclor^®^, a combination of cypermethrin, chlorpyrifos and piperonyl butoxide (PBO) (Ceva Animal Health) at the recommended concentration, plus 5 × and 10 × higher concentrations. The concentrations of deltamethrin were 0.25 mg/ml, 1.25 mg/ml and 2.5 mg/ml for the 1 ×, 5 × and 10 × concentrated doses, respectively. The concentrations of cypermethrin in the combination product were 0.56 mg/ml, 2.8 mg/ml and 5.5 mg/ml for the 1 ×, 5 × and 10 × concentrated doses, respectively. The concentrations of chlorpyrifos in the combination product were 0.94 mg/ml, 4.69 mg/ml and 9.94 mg/ml for the 1 ×, 5 × and 10 × concentrated doses, respectively. Not having used the same products for AIT and RIT is because both tests were developed according to protocols used in different laboratories. Although deltamethrin has a different excipient, the combination product was the same. The dilutions were made in a mixture of acetone/olive oil (2:1), and 0.9 ml was subsequently impregnated in triplicate into 10 × 7 cm Whatman (no.1) quality filter papers (Merck Life Science, Amsterdam). The filter papers were thoroughly dried in an aluminium tray in a fume hood for at least 60 min. The filter papers were subsequently grouped by concentration and sealed in plastic bags, kept in the dark at room temperature until used.

The larval progeny from the engorged females collected from each farm were utilised in the RIT. Each filter paper was folded half-width during the test to create a packet and sealed with bulldog clips. A tick-proof working station was prepared by lining it with water containing a 1% cleaning/disinfectant solution and securing double-sided tape around the edges as an additional barrier. Approximately 100 larvae were carefully transferred from the rearing vials to each packet using a fine brush. Control packets were loaded first, followed by the 1 × and 5 × packets, and finally, those containing the highest concentration of acaricide. The sealed larval packets were then incubated in an environmental chamber set to 27 ± 1 ºC with 80–90% relative humidity. After 24 h, each packet was opened, and the number of live and dead larvae was recorded, starting with the control group and progressing to the highest concentration. Larvae that were paralysed or exhibited movement only in their legs without the ability to walk were considered dead. The criterion for mortality was the larvae’s inability to walk after CO_2_ stimulation by gently breathing after the packets were opened. In total, three packets, each impregnated with a different concentration of acaricide, along with control packets, were prepared for each field isolate.

### Test read-out and statistical analysis

In the AIT, the following parameters were taken for each treated group: engorged females’ weight, egg-mass weight and percentage of larval hatch. The in vitro efficacy was calculated according to Drummond et al. using the following equations: (1) index of fertility (IF) = (egg-mass weight/engorged females weight) × % larval hatch; (2) in vitro efficacy (%) = 100 × [1 − (IF treated group/ IF control)]. Microsoft Excel was used to calculate the mean values and standard deviations for each parameter. An analysis of variance (ANOVA) followed by Tukey (*p* < 0.05) was used to compare the significance of differences in the IF for each treatment and the control. In the RIT, the percentage of mortality was recorded. The mean values and standard deviations for the mortality were calculated using Microsoft Excel, which was used to generate bar plots for comparison. Abbott’s formula was used to correct the mortality rate if the mortality rate in the control packets was 10% or less. The test was disregarded if mortality in the controls exceeded 10%. A population was considered resistant to the acaricides if the calculated in vitro efficacy (AIT) or mortality (RIT) was less than 90%. This mortality cut-off was based on the WAAVP guidelines recommendation for acaricide efficacy in the field [15]. A decision table was used to determine whether ticks were susceptible or carried a low, medium or high resistance level (Table 1). The mortality percentages of larvae exposed to 1 × recommended concentration of RIT and the in vitro efficacy obtained in the AIT were subjected to a Pearson correlation analysis using VassarStats (http://vassarstats.net/index.html).

**Table 1:** Decision table to determine the resistance level of ticks exposed to different concentrations of acaricides in the resistance intensity test (RIT)

1	A	Mortality in control ≤ 10%	Use Abbott’s formula for correction: continue at 2
	B	Mortality in control > 10%	Disregard this test and start again
2	A	Mortality at 1 × RD ≥ 90%	Susceptible
	B	Mortality at 1 × RD < 90%	Continue at 3
3	A	Mortality at 5 × RD ≥ 90%	Low resistance
	B	Mortality at 5 × RD < 90%	Continue at 4
4	A	Mortality at 10 × RD ≥ 90%	Moderate resistance
	B	Mortality at 10 × RD < 90%	High resistance

The 90% cut-off value is chosen on the basis of the WAAVP guidelines recommendation for the acaricide efficacy [15]

## Results

**Fig. 1:**
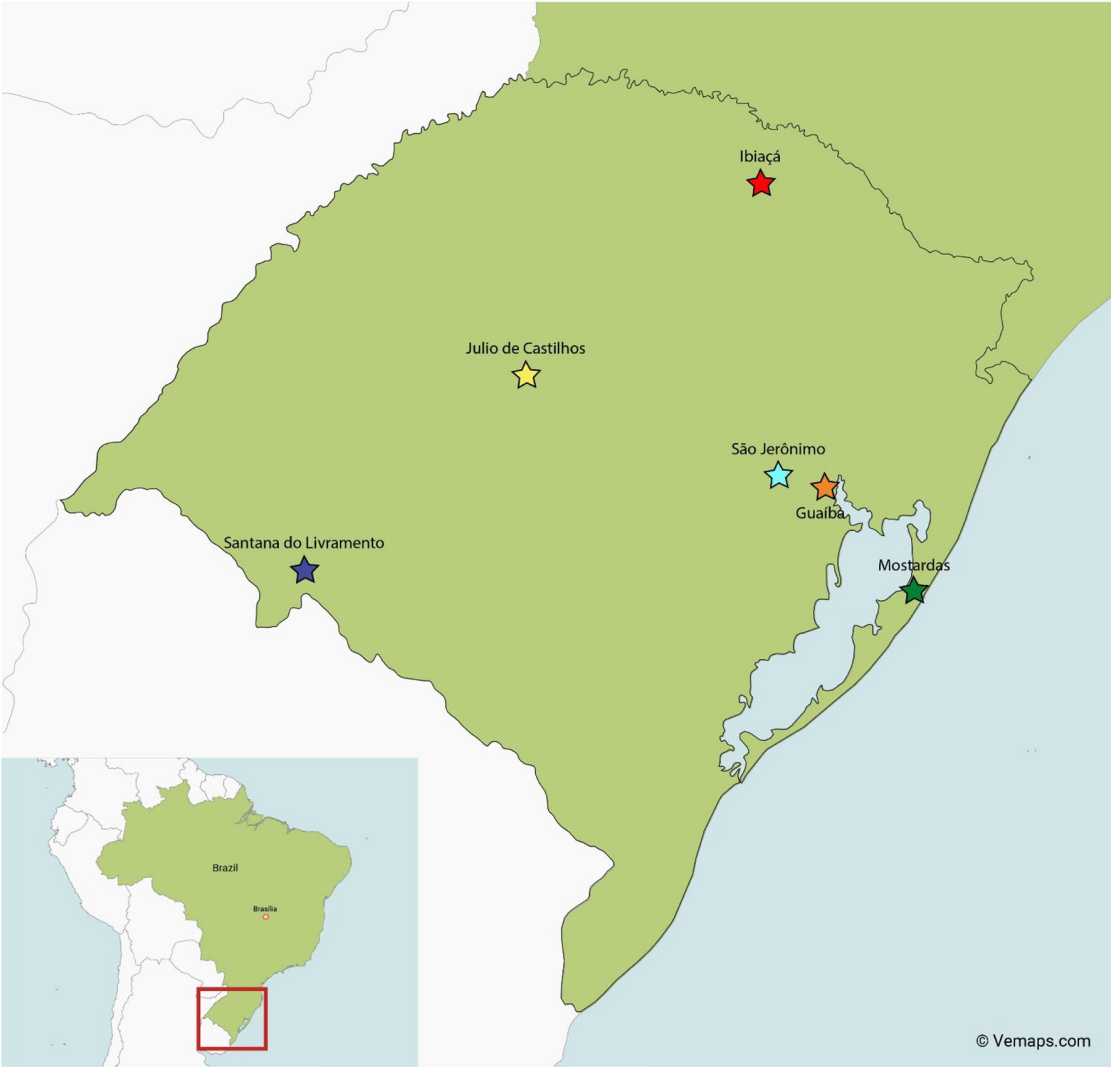
Geographical locations of ranches (*n* = 6) in Rio Grande do Sul state, Brazil, where adult *Rhipicephalus microplus* ticks were collected. Names alongside the symbols indicated the municipalities where sampling occurred

**Fig. 2:**
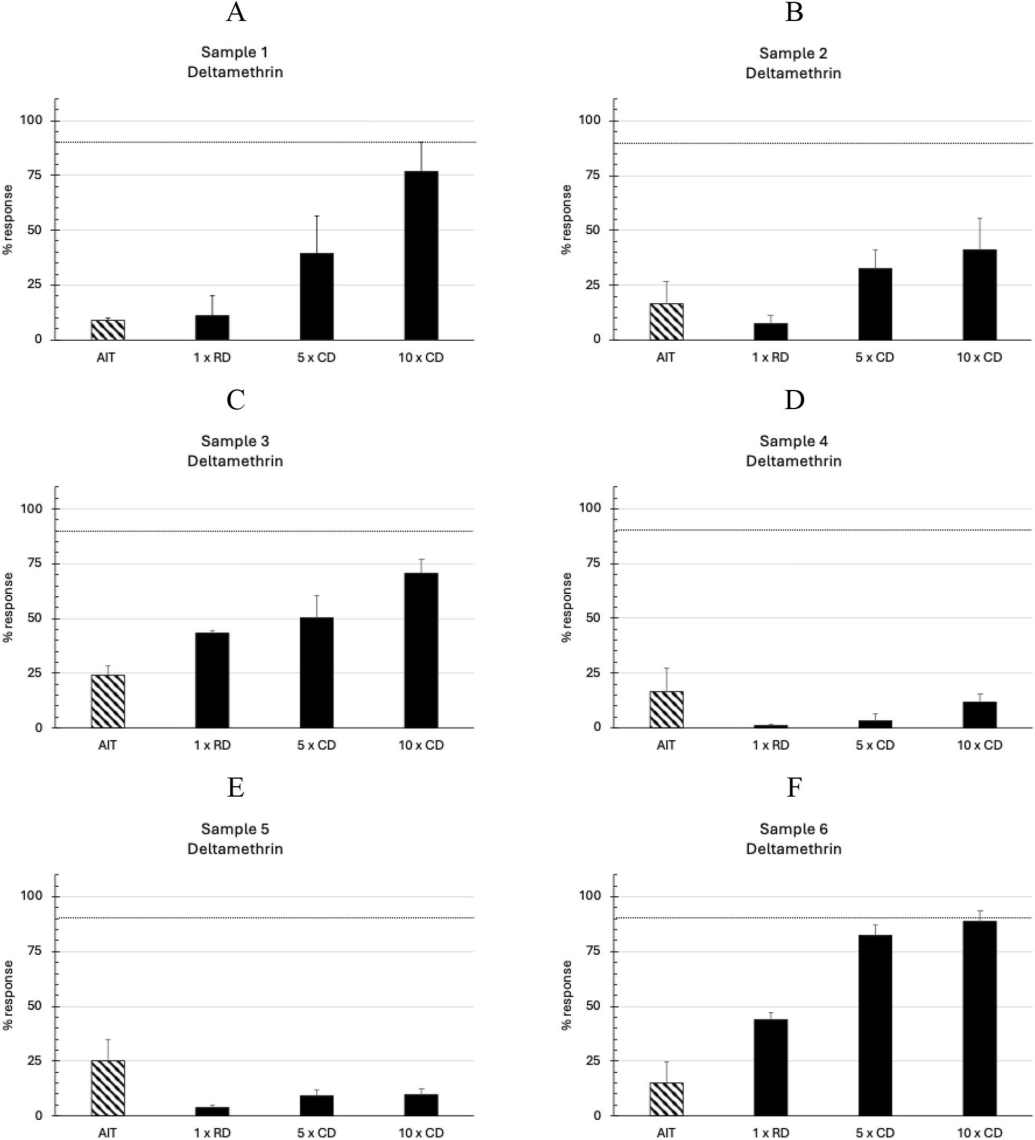
Results of adult immersion tests (AIT) and the resistance intensity test (RIT) against deltamethrin with Brazilian field strains of *Rhipicephalus microplus*. Panels A–F show field strains with high resistance. Bar plots represent the response to treatment as mean in vitro efficacy in adults and mean mortality of larvae in the RIT. *RD* recommended dose, *CD* concentrated dose. Error bars represent the standard deviations

**Fig. 3:**
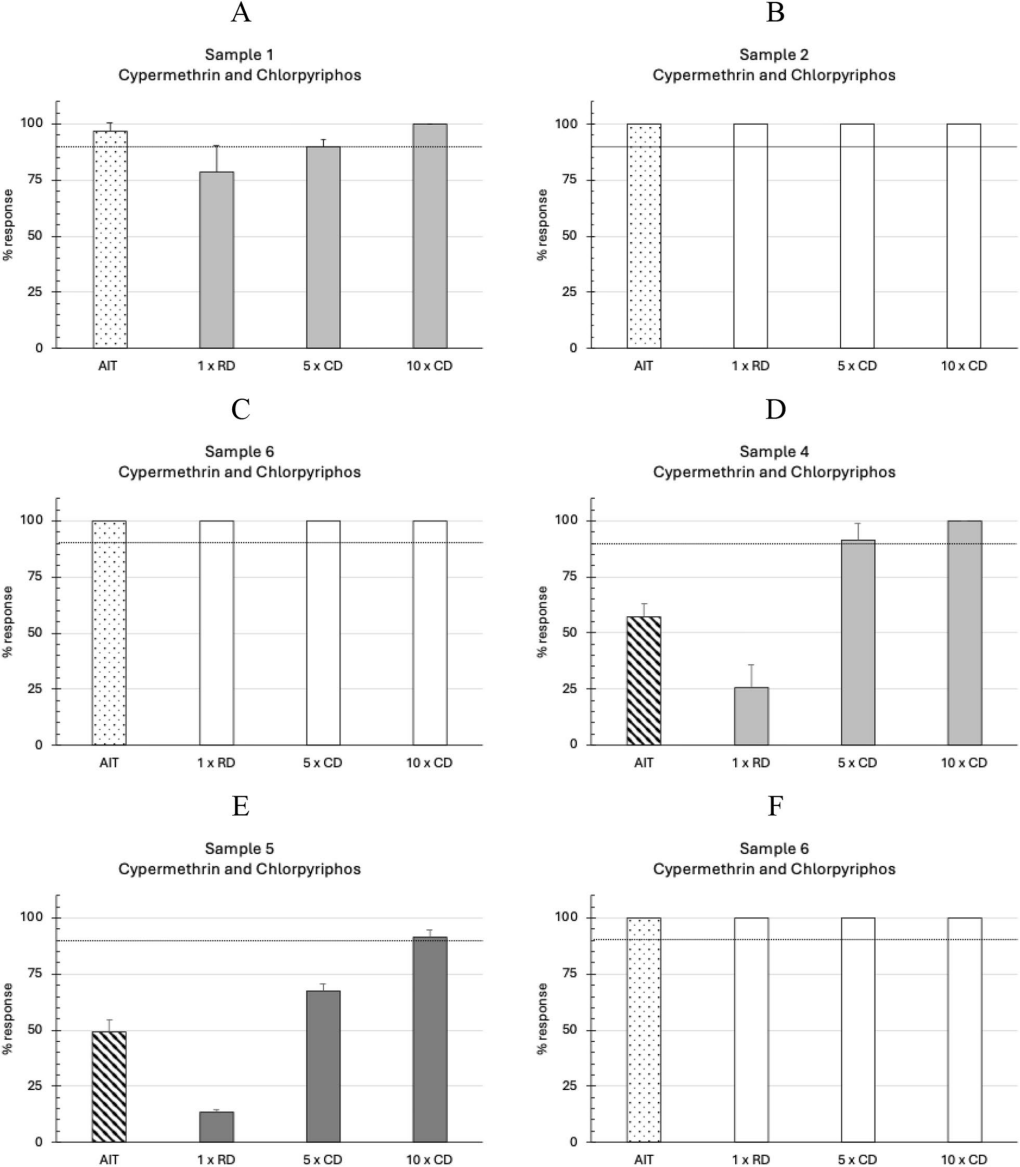
Results of adult immersion tests (AIT) and the resistance intensity test (RIT) against the combination of cypermethrin + chlorpyriphos + PBO with Brazilian field strains of *Rhipicephalus microplus*. Panels A and C show field strains with low resistance; panel D shows a field strain with moderate resistance; and panels B, E and F show susceptible strains. Bar plots represent the response to treatment as mean in vitro efficacy in adults and mean mortality of larvae in the RIT. *RD* recommended dose, *CD* concentrated dose. Error bars represent the standard deviations

**Fig. 4:**
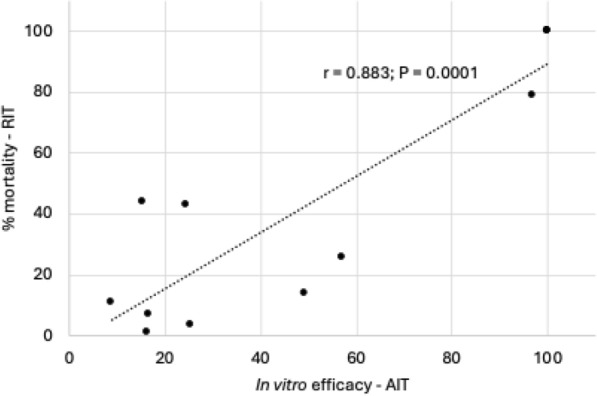
Relationship between the in vitro efficacy against adult females and mortality of larvae, exposed to the recommended concentrations of deltamethrin and the combination of cypermethrin and chlorpyriphos in the resistance intensity test (RIT) and the adult immersion test (AIT), *n* = 12 observations; *r* = Pearson product-moment correlation coefficient

The geographical locations of each ranch (*n* = 6) in the Rio Grande do Sul state of Brazil, where adult *R*. *microplus* ticks were collected, are indicated in Fig. 1. The in vitro efficacy of deltamethrin in the AIT ranged between 8.74% and 25.38% (Fig. 2, Table 2). According to the decision table, the RIT results on the larval progeny indicated a high level of resistance to deltamethrin in all the samples (Fig. 2; Table 1). The in vitro efficacy of the cypermethrin/chlorpyrifos/PBO combination in the AIT ranged between 49.31% and 100% (Fig. 3, Table 2). The corresponding RIT results on their larval progeny indicated a low level of resistance (Fig. 3, panels A and D) and a moderate resistance level (Fig. 3E). In contrast, three samples were susceptible to the combination of cypermethrin and chlorpyrifos in both tests—AIT and RIT (Fig. 3, panels B, C and F). The Pearson correlation coefficient (*r* = 0.883) indicated a high correlation between the test results of AIT and RIT at the recommended concentrations of both acaricides evaluated (Fig. 4).

**Table 2:** Results of the adult immersion tests with deltamethrin and the combination of cypermethrin/chlorpyriphos/PBO with field strains of *Rhipicephalus microplus* from Rio Grande do Sul, Brazil

Sample	Origin	Treatment group	Group of females weight (g)	Eggs weight (g)	Larval hatch (%)	Index of fertility	In vitro efficacy (%)
1	Ibiaçá	Control	2.17 (0.01)^a^	0.91 (0.04)	98.33 (2.89)	41.18 (2.26) b	–
		Deltamethrin	2.18 (0.01)	0.85 (0.06)	96.67 (2.89)	37.56 (1.68) b	8.74 (1.5)
		Cypermethrin/chlorpyriphos	2.18 (0.01)	0.10 (0.04)	21.67 (20.21)	1.19 (1.42) c	96.83 (3.86)
2	São Jerônimo	Control	2.22 (0.01)	1.05 (0.08)	96.67 (2.89)	45.94 (3.84) b	–
		Deltamethrin	2.21 (0.01)	0.94 (0.03)	90 (5)	38.13 (1.85) c	16.48 (10.01)
		Cypermethrin/chlorpyriphos	2.22 (0.01)	0 (0)	0 (0)	0 (0) d	100 (0)
3	Julio de Castilhos	Control	2.04 (0.01)	1.04 (0.1)	95 (5)	48.64 (6.61) b	–
		Deltamethrin	2.04 (0.01)	0.85 (0.09)	88.33 (2.89)	36.61 (3.08) c	24.34 (4.32)
		Cypermethrin/chlorpyriphos	2.04 (0.01)	0 (0)	0 (0)	0 (0) d	100 (0)
4	Guaíba	Control	2.33 (0.05)	1.11 (0.12)	96.67 (5.77)	46.35 (7.78) b	–
		Deltamethrin	2.37 (0.01)	0.99 (0.05)	91.67 (2.89)	38.20 (1.77) b	16.39 (10.66)
		Cypermethrin/chlorpyriphos	2.37 (0.01)	0.61 (0.07)	65 (13.23)	16.44 (2.63) c	56.99 (6.13)
5	Santana do Livramento	Control	2.25 (0.01)	0.97 (0.2)	98.33 (2.89)	42.33 (8.06) b	–
		Deltamethrin	2.25 (0.01)	0.79 (0.25)	91.67 (2.89)	32.00 (9.26) b, c	25.38 (9.52)
		Cypermethrin/chlorpyriphos	2.24 (0.01)	0.39 (0.13)	95 (5)	16.55 (6.13) c	49.31 (5.30)
6	Mostardas	Control	2.47 (0.01)	1.16 (0.07)	98.33 (2.89)	46.34 (1.94) b	–
		Deltamethrin	2.45 (0.01)	0.98 (0.08)	98.33 (2.89)	39.20 (4.11) c	15.29 (9.48)
		Cypermethrin/chlorpyriphos	2.46 (0.01)	0 (0)	0 (0)	0 (0) d	100 (0)

^a^Standard deviation; b, c, d: different letters represent significant differences on the IF calculated among the groups within the test with each sample

## Discussion

Acaricide resistance in cattle ticks is a significant concern in tropical and subtropical regions, particularly Brazil. This study improved the relationship between bioassay test results and actions to mitigate acaricide resistance in the field. As a primary bioassay, the AIT provided relevant information on the presence of resistance by immersing engorged female ticks in a solution at the recommended dose of an acaricide.

The observation that the adult *R*. *microplus* ticks resisted a recommended dose of deltamethrin in the AIT (Fig. 1) and were able to produce a progeny (Table 2) demonstrated the presence of resistance to synthetic pyrethroids but not the level of resistance. However, the corresponding RIT results on their larval progeny indicated a high resistance level (Fig. 2), which could lead to discontinuing synthetic pyrethroids at these cattle ranches (Fig. 1).

Moreover, it was observed that some adult *R*. *microplus* ticks also resisted the recommended dose of cypermethrin/chlorpyrifos/PBO in the AIT (Fig. 3) and were able to produce progeny (Table 2; samples 1, 4 and 5), demonstrating the presence of resistance, but again not the level of resistance. The corresponding RIT results on their larval progeny indicated a similar response ranging from fully susceptible to low or moderate resistance. This suggests that organophosphates, including chlorpyrifos and chlorfenvinphos, used in many combination products with active ingredients from other acaricidal classes, may control synthetic pyrethroid-resistant tick populations. Furthermore, the Pearson correlation coefficient (*r* = 0.883) revealed a high correlation between both tests (Fig. 4).

This paper presents the resistance intensity test (RIT) by adopting a similar protocol from the WHO guidelines for resistance detection in malaria mosquitoes [16]. In addition to reducing the number of test papers and larval ticks, using the recommended concentration of acaricide and commercially available products rather than technical grade drugs for impregnating filter papers created a direct link to acaricidal products available on the market. This protocol uses the recommended dose of the acaricide (1×), plus 5 × and 10 × higher concentrations, revealing low, moderate and high resistance intensity. The choice to use high concentrations (5 × and 10 ×) in the resistance intensity test (RIT) is aimed at quantifying the upper limits of resistance, which provides valuable insights into the intensity and potential severity of acaricide resistance in a population. While it is true that these concentrations may indicate that a product would be ineffective at those levels in the field, they offer a clear threshold for understanding resistance escalation beyond common therapeutic doses. This allows us to assess whether resistance is emerging at moderate levels or has reached a point where standard treatments are unlikely to work, which is critical for developing long-term management strategies.

The complexity of determining resistance intensity, whether through full dose–response bioassays, such as the LPT and LIT’s, calculated resistance ratios, underscores the need for careful interpretation. In this study, the aim was not only to detect resistance but also to quantify its severity. This information is essential for designing rotation schemes or integrating alternative control measures in cases where high resistance levels might render standard treatment protocols ineffective.

While RIT results may only directly translate into therapeutic efficacy in animals with field verification, they provide an important first step in resistance surveillance. We can guide farmers on when to avoid certain products or switch to alternatives by identifying significant resistance intensities. The next step would be linking these laboratory findings to field efficacy studies, thus making the RIT a valuable tool for risk assessment and management recommendations in acaricide resistance.

This study aimed to present a novel bioassay for measuring the intensity of acaricide resistance. The current study’s limitation is the number of sampled cattle farms and the further validation of the results compared with the in vivo efficacy of the acaricides in the field. Also, the correlation between the RIT and the resistance ratio (RR) obtained in classical LPT assays deserves further investigation. Finally, it is further investigated whether the RIT can be used for a broader range of acaricidal classes.

## Conclusions

This study demonstrates the value of integrating the adult immersion test (AIT) with the resistance intensity test (RIT) in managing acaricide resistance. By screening adult ticks with AIT and applying RIT on their larval progeny, farmers can gain quantitative information on the intensity of resistance. This approach not only refines the detection of resistance but also enables more informed and assertive decisions regarding which acaricide products to use in the field. Consequently, this strategy can lead to more effective tick control measures and potentially slow the spread of resistance.

The findings underscore the importance of using both tests in tandem, particularly in regions with high acaricide usage. Future research should focus on refining these methods and exploring their application in large-scale field conditions. Incorporating these tests into routine monitoring protocols will significantly enhance long-term acaricide resistance management, safeguarding animal health and productivity.

## Data Availability

No datasets were generated or analysed during the current study.

## Abbreviations

AIT: Adult immersion test
LPT: Larval packet test
RIT: Resistance intensity test
PBO: Piperonyl butoxide
